# *Drosophila* CRISPR/Cas9 mutants as tools to analyse cardiac filamin function and pathogenicity of human FLNC variants

**DOI:** 10.1242/bio.059376

**Published:** 2022-09-16

**Authors:** Flavie Ader, Maria Russi, Laura Tixier-Cardoso, Estelle Jullian, Elodie Martin, Pascale Richard, Eric Villard, Veronique Monnier

**Affiliations:** 1 APHP, Hôpital Universitaire Pitié-Salpêtrière, Département Médico-Universitaire BioGEM, UF Cardiogénétique et Myogénétique, Service de Biochimie Métabolique, F-75013 Paris, France; 2 Sorbonne Université, INSERM UMRS 1166 and ICAN Institute, F-75013 Paris, France; 3 Unité Pédagogique de Biochimie-Département des Sciences Biologiques et Médicales, UFR de Pharmacie-Faculté de Santé, Université Paris Cité, Paris, France; 4 Université Paris Cité, Unité de Biologie Fonctionnelle et Adaptative-BFA, UMR 8251, CNRS, F-75013 Paris, France

**Keywords:** Filamin, Cardiomyopathy, Cheerio, Heart function, *Drosophila* model

## Abstract

Filamins are large proteins with actin-binding properties. Mutations in *FLNC*, one of the three filamin genes in humans, have recently been implicated in dominant cardiomyopathies, but the underlying mechanisms are not well understood. Here, we aimed to use *Drosophila melanogaster* as a new *in vivo* model to study these diseases. First, we show that adult-specific cardiac RNAi-induced depletion of *Drosophila* Filamin (dFil) induced cardiac dilatation, impaired systolic function and sarcomeric alterations, highlighting its requirement for cardiac function and maintenance of sarcomere integrity in the adult stage. Next, we introduced in the *cheerio* gene, using CRISPR/Cas9 gene editing, three missense variants, previously identified in patients with hypertrophic cardiomyopathy. Flies carrying these variants did not exhibit cardiac defects or increased propensity to form filamin aggregates, arguing against their pathogenicity. Finally, we show that deletions of the C-term part of dFil carrying the last four Ig-like domains are dispensable for cardiac function. Collectively, these results highlight the relevance of this model to explore the cardiac function of filamins and increase our understanding of physio-pathological mechanisms involved in FLNC-related cardiomyopathies.

## Abstract (precis)

**Summary:** CRISPR mutants of *Drosophila* filamin, shown here to be required for adult cardiac function and sarcomeric integrity, revealed that the C-term region is dispensable and opens ways to study human variants.

## INTRODUCTION

Filaminopathies are a group of diseases linked to variants in genes encoding filamins (FLNs), a family of large actin-binding proteins with actin-crosslinking properties. This family is composed in humans of three proteins (FLNA, FLNB, and FLNC) encoded by different genes and exhibiting 60-80% overall amino acid identity (Chakarova et al., 2000). *FLNA* and *FLNB* are both widely expressed and pathogenic variants in *FLNA* have been reported in several neurological syndromes and disorders affecting conjunctive tissues (Wade et al., 2020), whereas *FLNB* variants have mainly been involved in skeletal disorders (Krakow et al., 2004). *FLNC* is predominantly expressed in skeletal and cardiac muscles (Thompson et al., 2000) and variants in this gene were first involved in the development of dominant distal and myofibrillar myopathies. These diseases develop during adulthood and are associated with a cardiomyopathy in about 30% of cases (Duff et al., 2011). Variants have also been described as responsible of dominant adult isolated cardiomyopathies (Brodehl et al., 2016; Ortiz-Genga et al., 2016; Valdés-Mas et al., 2014). Whereas myofibrillar myopathies are mainly due to missense variants (Fürst et al., 2013; Verdonschot et al., 2020), truncating variants, missense and inframe ins-del have been described in cardiomyopathies (Ader et al., 2019; Verdonschot et al., 2020).

FLNC, as other filamins, is composed of an N-terminal actin-binding domain followed by immunoglobulin-like (Ig) repeats, the 24th and last repeat located at the C-terminus being involved in homodimerization. Ig repeats are subdivided in two domains, the Rod1 domain and the more globular Rod2 domain, susceptible to unfold in response to mechanical force. FLNC is localised at the sarcomeric Z disc, the sarcolemma, intercalated discs in mammalian cardiac muscle and myotendinous junctions in skeletal muscle (van der Ven et al., 2000). At the Z-disc, FLNC interacts by its Rod2 domain with a large number of partners including myotilin, FATZ-1, titin and myopodin (Gontier et al., 2005; Labeit et al., 2006; Linnemann et al., 2010; van der Ven et al., 2000). *In vitro* and *in vivo* models have been developed in order to understand the normal function of FLNC as well as the mechanisms of pathogenicity in FLNC-related diseases. Mice expressing a truncated FLNC lacking the last four Ig repeats exhibit a severe muscular phenotype with a reduced number of muscle fibers and primary myotubes, highlighting the role of FLNC in primary myogenesis (Dalkilic et al., 2006). FLNC was also shown to be rapidly recruited to sites of myofibril repair, both in cultured cardiomyocytes and *in vivo* in Zebrafish, suggesting its involvement in the maintenance of myofibrillar integrity (Leber et al., 2016). This is further supported by the phenotypes observed in the medaka mutant *zacro*. This mutant fish, that bears a homozygous nonsense mutation (*zac*, K1680X, homozygous) in the *flnc* gene leading to a premature termination and a strong reduction of *flnc* transcripts, presents muscle degeneration that is alleviated by inhibition of muscle contraction, suggesting that FLNC is required for maintenance of muscle structural integrity in response to mechanical stress (Fujita et al., 2012).

Removal of damaged FLNC from the Z-disc and its subsequent degradation is ensured by chaperone-assisted selective autophagy (CASA), a mechanism involving ubiquitin-dependent clearance through lysosomal degradation. Accordingly, in muscles of mice deficient for the lysosomal associated membrane protein LAMP2, the resulting inhibition of autophagy induces filamin accumulation and aggregation (Arndt et al., 2010). Several variants of *FLNC* identified in patients with myofibrillar myopathies are prone to aggregate, leading to disintegration of myofibrils (Löwe et al., 2007; Vorgerd et al., 2005). Indeed, the human FLNC variant W2710X, associated with myofibrillar myopathy and leading to a shorter protein without the dimerization domain, formed protein aggregates when expressed in zebrafish. Surprisingly, beside these aggregative properties, this variant was also able to localise correctly at the Z-disk and to rescue the fiber disintegration phenotype when expressed in zebrafish depleted for endogenous FLNC (Ruparelia et al., 2016). Thus, this variant appears at least partially functional, suggesting a pathogenic mechanism involving toxic effects of aggregates. In addition, expression in rat cardiomyoblasts of three FLNC variants carried by patients with hypertrophic cardiomyopathy led to the formation of large filamin aggregates (Valdés-Mas et al., 2014).

While the first description of *FLNC* variants associated with isolated cardiomyopathy is recent (2014), a large number of *FLNC* variants have since been reported. The prevalence of this gene is estimated to be about 1-5% in patients with dilated cardiomyopathy (DCM) and between 1 to 9% in patients with hypertrophic cardiomyopathy (HCM) (Ader et al., 2019; Begay et al., 2016; Cui et al., 2018; Janin et al., 2017; Ortiz-Genga et al., 2016; Valdés-Mas et al., 2014). An arrhythmogenic trait has been observed in FLNC-related DCM, since frequent personal or familial history of sudden cardiac death have been described (Ader et al., 2019; Begay et al., 2016). As left ventricular non-compaction and restrictive cardiomyopathies are rare phenotypes, the prevalence remains less studied but some FLNC variants have been published (Ader et al., 2019; Brodehl et al., 2016; Tucker et al., 2017). Regarding the nature of these variants, truncating mutations have been recently considered with definite proof of implication in the development of DCM (Jordan et al., 2021). Variants identified in HCM patients are mainly missense with a preferential localisation in the Rod2 domain (Ader et al., 2019; Verdonschot et al., 2020). However, in most cases, their pathogenicity has not been demonstrated, and the physio-pathological mechanisms, which could involve either loss or toxic gain of function associated or not with aggregation, are largely unknown.

The aim of this study was to evaluate the relevance of using *Drosophila melanogaster* as a new animal model to study FLNC-related cardiomyopathies. This organism has emerged as an alternative model to mammals to study cardiomyopathies, taking advantage of large gene conservation of human disease-related genes, the availability of powerful genetic tools and the opportunity to conduct experiments on large populations in a relatively short time. Indeed, *Drosophila* models of a large number of cardiac diseases have now been reported (Taghli-Lamallem et al., 2016). *Drosophila* filamin (dFil) is encoded by a single gene called *cheerio* (*cher*). dFil shares 45% of amino-acid identity with human FLNC. It has initially been involved in the formation of the ring canal during oocyte development (Robinson et al., 1997; Sokol and Cooley, 1999) and was also shown to be essential in the nervous system for motor neuron growth cone guidance (Lee and Schwarz, 2016) and the function of long-term memory (Bolduc et al., 2010). In muscles, dFil interacts with Salimus (sls), the *Drosophila* ortholog of titin, and dFil depletion results in sarcomeric defects (Brooks et al., 2020; González-Morales et al., 2017; Wojtowicz et al., 2015). The function of dFil in *Drosophila* heart has not been yet studied.

Here, we first describe the impact of RNAi-induced dFil depletion on cardiac function and cardiomyocyte morphology. We observed cardiac dilatation and contractile deficits associated with strong sarcomeric disorganisation, observable even when dFil depletion was induced only at the adult stage. Next, we evaluated the pathogenicity of three missense FLNC variants previously identified in patients with HCM and for which pathogenicity was not assessed (Ader et al., 2019), but failed to detect any functional cardiac phenotype or increased propensity to aggregate in flies. Finally, we analysed dFil mutants in which the last four Ig-like domains were deleted. We did not observe any functional and histological defects showing that the C-term putative dimerization domain is dispensable for cardiac function.

## RESULTS

### Cardiac depletion of dFil leads to heart dilatation and impaired systolic function

**Fig. 1.**
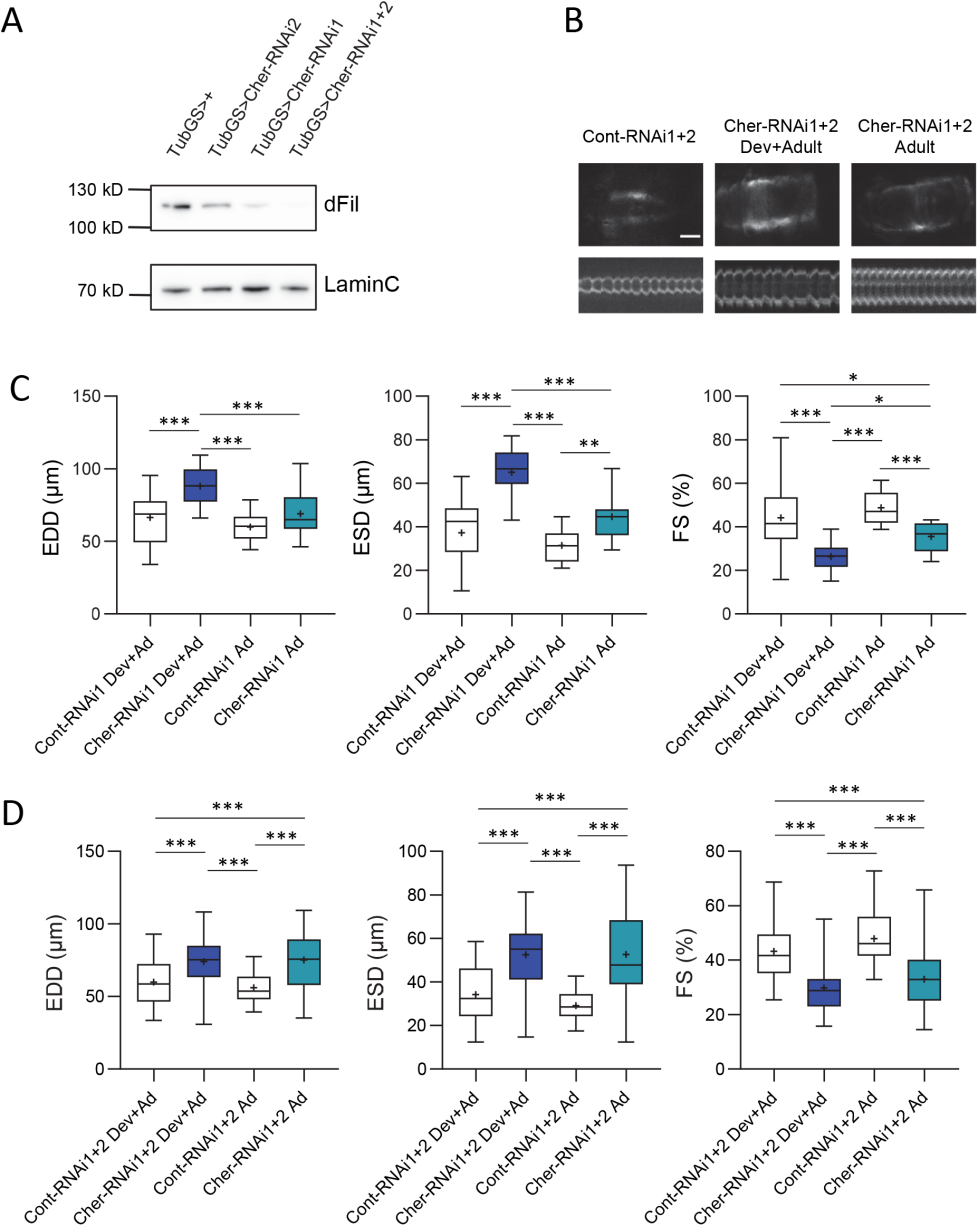
**RNAi-mediated dFil depletion leads to cardiac dilatation and impaired systolic function.** (A) The level of expression of dFil in *tubGS>UAS-cherRNAi* flies compared to *tubGS>+* control was analysed by western blot (WB), with antibody directed against LaminC as a loading control. All flies were fed with RU486 (100 µg ml^−1^) only during adulthood and analysed at 9 days of age. (B) Anterior part of hearts (abdominal segments A1/A2, observed through the cuticle under ultraviolet light) and representative M-Modes (generated by horizontal alignment of rows extracted at the same position for each movie frame) of *Hand-GS>UAS-mitoGFP, UAS-Cont-RNAi1+2* and *Hand-GS>UAS-mitoGFP, UAS-Cher-RNAi1+2* flies fed with RU486 (10 µg ml^−1^) during development and adulthood (Dev+Ad) or only during adulthood (Ad). Scale bar: 50 µm. (C) End-Diastolic Diameters (EDD, µm), End-Systolic Diameters (ESD, µm) and Fractional Shortening (FS, %) of *Hand-GS>UAS-mitoGFP, UAS-Cont-RNAi1* flies (Cont-RNAi1 Dev+Ad: *n*=19, Ad: *n*=17) and *Hand-GS>UAS-mitoGFP, UAS-Cher-RNAi1* flies (Cher-RNAi1 Dev+Ad: *n*=24, Ad: *n*=17). (D) EDD, ESD and FS of *Hand-GS>UAS-mitoGFP, UAS-Cont-RNAi1+2* flies (Cont-RNAi1+2, Dev+Ad: *n*=14, Ad: *n*=10) and *Hand-GS>UAS-mitoGFP, UAS-Cher-RNAi1+2* flies (Cher-RNAi1+2 Dev+Ad: *n*=37, Ad: *n*=14). All cardiac imaging experiments were performed on 12–13-day-old male flies. Flies were treated with RU486 (10 µg ml^−1^) continuously (Dev+Ad) or only during adulthood (Ad). Significant differences are indicated: * *P*<0.05, ** *P*<0.01, *** *P*<0.001.

To investigate the effects of cardiac-specific depletion of dFil, we downregulated the expression of *cher* by RNA interference in *Drosophila* flies. We used two UAS-cher-RNAi constructs, carried by the TRIP *cher^JF0277^* and the VDRC *cher^KK107451^* lines, named below Cher-RNAi1 and 2 respectively. The ability of these constructs to efficiently decrease the dFLN level was previously reported (González-Morales et al., 2017) and was further assessed here in whole flies using a tub-GS RU486-inducible ubiquitous driver. Partial depletion was observed in flies carrying a single RNAi construct and the protein level was further reduced in flies carrying both RNAi constructs (Fig. 1A). Expression of UAS-Cher-RNAi was then driven by the heart-specific Geneswitch driver Hand-GS. The activity of this driver is dependent of RU486 that can be added to the fly food either during both development and adulthood or specifically during adulthood, to study and compare continuous versus adult-specific dFil depletion. The driver was also combined to a UAS-mitoGFP construct, to express a mitochondrial GFP specifically in cardiomyocytes, allowing heart labelling and high-speed video recording of cardiac beats *in vivo* on anaesthetised flies as previously described. Representative pictures of *Drosophila* heart (end diastolic time) and M-mod obtained from movies showed diastolic enlargement of the cardiac tube in RNA1+2 compared with the control (Fig. 1B) (Monnier et al., 2012; Palandri et al., 2018; Tricoire et al., 2014). Appropriate controls were used, namely flies carrying a TRIP (UAS-Cont-RNAi1) or a VDRC (UAS-Cont-RNAi2) construct, that both target genes that are not expressed in the heart. All the *Hand-GS>UAS-mitoGFP, UAS-Cont-RNAi* control flies, carrying a single Cont-RNAi or the combination of both, exhibited similar cardiac functional parameters, end-diastolic diameters (EDD), end-systolic diameters (ESD) and fractional shortening (FS), following continuous or adult-restricted RU-486 treatment (Fig. S1). When *Hand-GS>UAS-mitoGFP, UAS-cher-RNAi* flies were treated with RU-486 from the development stage, they exhibited cardiac dilatation as revealed by EDD that were 33% and 31% higher than controls for RNAi1 and RNAi2, respectively (Fig. 1C; Fig. S2). ESD were also strongly increased, by 79% for RNAi1 and 77% for RNAi2 flies. This shows that systolic function is affected, as further confirmed by the strong decrease in FS. Then, we treated flies with RU-486 only during adulthood. In these conditions, EDD was not significantly increased in flies carrying only one RNAi and ESD was moderately increased, by 14% with the RNAi1 construct (Fig. 1C; Fig. S2). Next, we measured the cardiac functional parameters of flies carrying both RNAi constructs, and observed increased EDD and ESD as well as a decreased FS with similar values between continuous or adult-restricted RU486 treatments (Fig. 1B,D). These results indicate that dFil depletion at the adult stage leads to cardiac dilatation and altered systolic function.

### Cardiac depletion of dFil leads to sarcomeric disorganisation

**Fig. 2.**
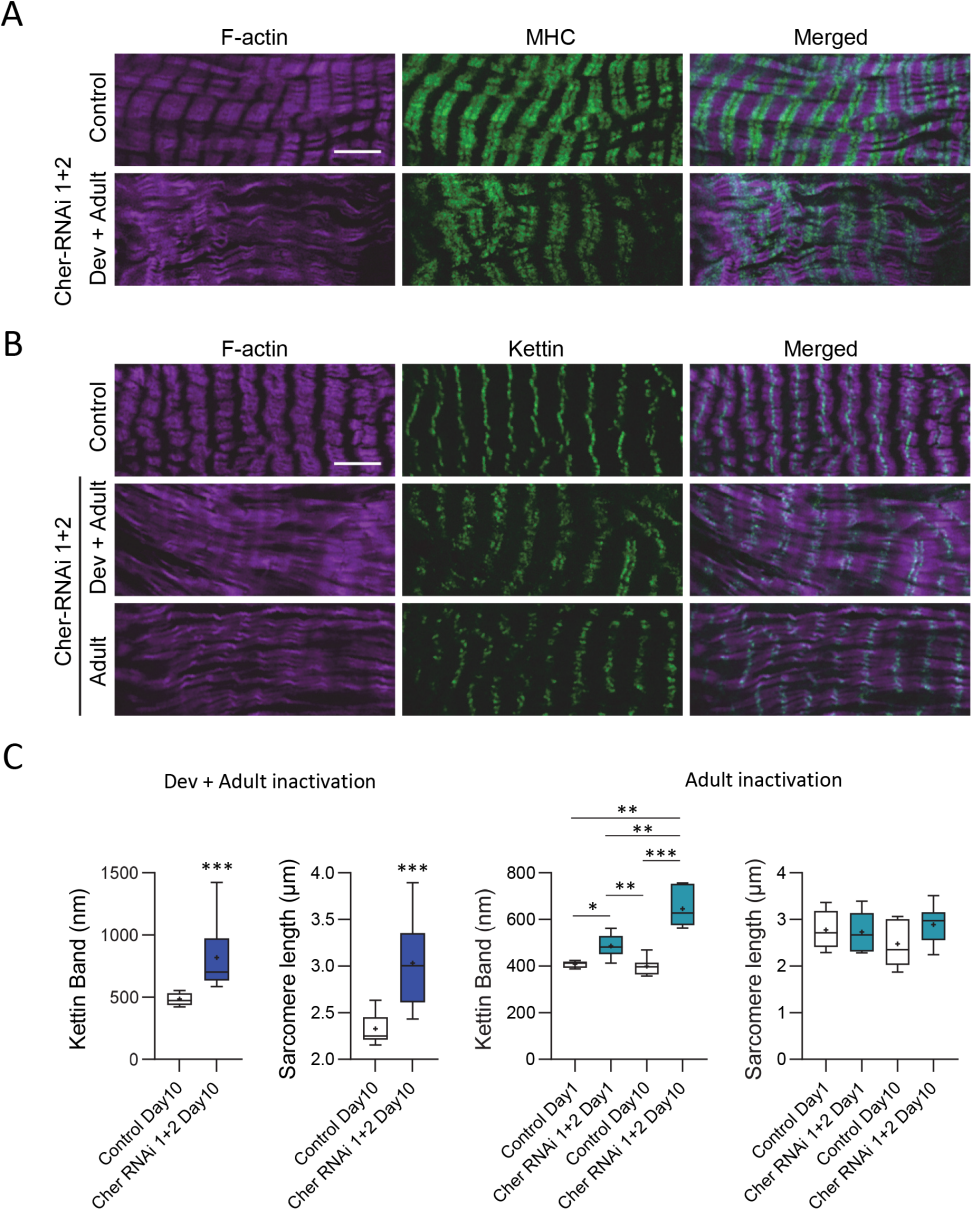
**RNAi-mediated dFil depletion results in sarcomeric defects.** (A) Hearts of 10-day-old adult *Hand-GS>UAS-cher-RNAi1+2, MHC:GFP* and *Hand-GS>MHC:GFP* control male flies were dissected and double-labelled with phalloidin to stain F-actin and an anti-GFP antibody to stain the MHC-GFP fusion protein. All flies were fed with RU486 during both development and adulthood (10 µg ml^−1^). Scale bar: 5 µm. (B) Hearts of 10-day-old adult *Hand-GS>UAS-cher-RNAi1+2* and *Hand-GS>+* control male flies were dissected and double-labelled with phalloidin to stain F-actin and an anti-Kettin antibody. Flies were fed with RU486 during both development and adulthood or only during adulthood (10 µg ml^−1^). (C) Quantification of the width of the kettin band and sarcomere lengths in hearts of 10-day-old flies treated during development and adulthood (left panel: *Hand-GS>+*: *n*=9, *Hand-GS>UAS-cher-RNAi1+2*: *n*=10) or in hearts of flies treated only during adulthood (right panel: *Hand-GS>+*: 1 day *n*=6, 10 days *n*=7, *Hand-GS>UAS-cher-RNAi1+2*: 1 day *n*=7, 10 days *n*=7.) Significant differences are indicated: * *P*<0.05, ** *P*<0.01, *** *P*<0.001.

We also characterised the structural defects induced by dFil depletion in cardiomyocytes. To this purpose, we first used a protein trap line with GFP sequence inserted in the gene encoding the myosin heavy chain gene (MHC) (Morin et al., 2001). We characterised sarcomeric organisation in cardiomyocytes of *Hand-GS>UAS-cher-RNAi1+2, MHC:GFP* flies compared to *Hand-GS>MHC:GFP* flies, following developmental and adult RU486 treatment (Fig. 2A). Whereas myosin striations associated with actin fibers were present, the periodic F-actin striations were fully absent in cardiomyocytes of dFil-depleted hearts. Then, we performed Kettin staining (the 500kD isoform of Sls/Titin) to stain the Z-band. When dFil depletion was induced during the developmental and at the adult stage, we observed a widening of the kettin band of 69%, associated with an increase of sarcomere length of 30% (Fig. 2B,C). A general view of the conical chamber also allowed us to visualise its enlargement associated with abnormalities of the sarcomeric organisation (Fig. S3).

When dFil depletion was induced only during adulthood, we observed a progressive widening of the kettin band, which was only 19% larger than controls in 1-day-old adults but 61% larger in 10-day-old adults (Fig. 2B,C). Interestingly, this was not associated with modifications on the sarcomere length. This suggests that the widened Z-disc phenotype induced by filamin depletion is not a consequence of a global increase in sarcomere length but rather an earlier step in the alteration of sarcomere integrity.

### Evaluation of the pathogenicity of three hFLNC missense variants

**Fig. 3.**
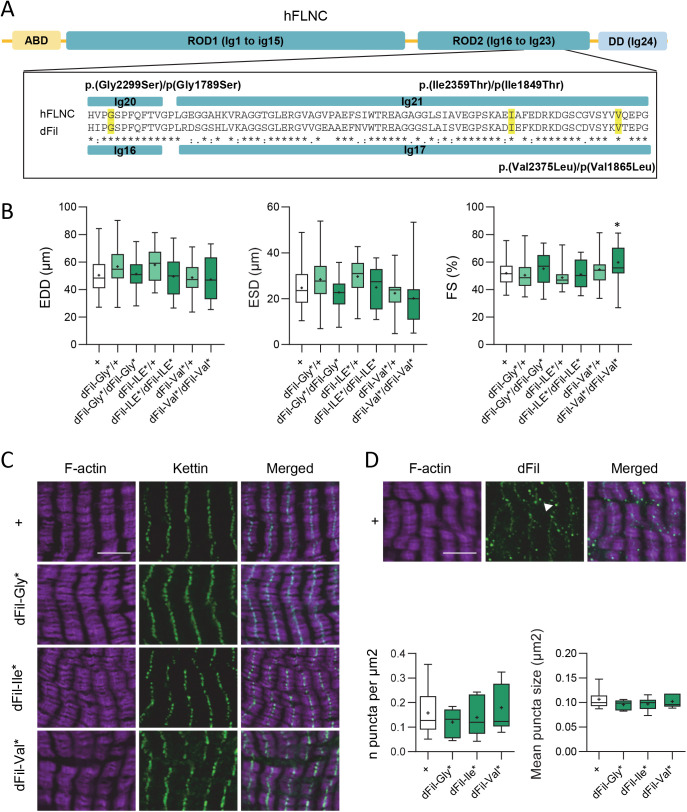
**Flies carrying human FLNC variants do not exhibit cardiac functional or structural defects and are not increasingly prone to aggregation.** (A) Schematic diagram of human FLNC with a focus on Immunoglobin-like domains 20 and 21 in which the three selected missense variants are located. Sequence alignment with *Drosophila* dFil is shown. ABD: actin binding domain; DD: dimerization domain. (B) End-diastolic diameters (EDD, µm), end-systolic diameters (ESD, µm) and fractional shortening (FS, %) of 23–25-day-old *Hand-GS>UAS-mitoGFP controls* (+, *n*=69), Hand-GS>UAS-mitoGFP, dFil-Gly*/+ (*n*=48) or dFil-Gly*/ dFil-Gly* (*n*=22), Hand-GS>UAS-mitoGFP, dFil-Ile*/+ (*n*=27) or dFil-Ile*/ dFil-Ile* (*n*=17) and *Hand-GS>UAS-mitoGFP*, dFil-Val*/+ (*n*=32) or dFil-Val*/ dFil-Val* (*n*=19) male flies. All values are means (±s.e.m.). Significant differences are indicated: * *P*<0.05, ** *P*<0.01. (C) Hearts of 10-day-old adult control (*w^1118^*) and homozygous dFil-Gly*, dFil-Ile* and dFil-Val* male flies were dissected and double-labelled with phalloidin to stain F-actin and with an anti-Kettin antibody. Scale bar: 5 µm. (D) Hearts of 10-day-old adult control (*w^1118^*, *n*=7) and homozygous dFil-Gly* (*n*=7), dFil-Ile* (*n*=6) and dFil-Val* (*n*=7) male flies were dissected and double-labelled with phalloidin to stain F-actin and with anti-dFil antibody. The number and mean sizes of aggregate-like dense bodies interspersed between actin filaments (white arrowhead) were quantified and do not show significant differences between genotypes. Scale bar: 5 µm.

Next, we aimed to use the *Drosophila* model to assess the pathogenicity of human FLNC variants identified in patients affected by cardiomyopathies. This approach is made possible by the gene-editing tools offered by the CRISPR/Cas9 system and is relevant here because of the high level of conservation of filamin between *Drosophila* and humans. We selected three variants identified in a cohort of 700 HCM patients (Ader et al., 2019). The selection criteria were the location in the Rod2 domain in which missense variants associated to HCM were clustered, the low frequency in the control database (GnomAD) and the conservation of affected amino acids in flies. The three variants are Gly2299Ser, Ile2359Thr and Val2375Leu, located in Ig20 and 21 of the Rod2 domain (Fig. 3A). The septum hypertrophy in these patients was mild, with interventricular septum thickness ranging from 13.8 to 17 mm and an age at diagnosis ranging from 16 to 49 years (Ader et al., 2019). We modified by CRISPR/Cas9 gene editing the corresponding amino acids in dFil, namely Gly1789Ser (in Ig16), Ile1849Thr and Val1865Leu (both in Ig17) (Fig. 3A; Fig. S4). These knock-in (KI) lines, named below dFil-Gly*, dFil-Ile* and dFil-Val*, were all viable at the homozygous state. We analysed cardiac function of flies (23-25 days old) carrying the variants either at the heterozygous state, to mimic the patient situation, or at the homozygous state (Fig. 3B). We failed to identify any significant differences in EDD between all these genotypes. ESD and FS were also not significantly different between the variants and control flies, except for dFil-Val* for which FS was even slightly higher than in controls. Thus, these three variants do not affect cardiac function in flies, neither at the heterozygous nor at the homozygous state. To go further, we also performed immunostaining to observe sarcomeric organisation. The actin network along with the distribution of the kettin signal at the Z-disc were similar to control flies (Fig. 3C). Then we performed additional experiments on older flies, and we didn't observe functional or structural defects at 5 weeks of age (Fig. S5).

As mentioned previously, aggregation of FLNC variants have been observed in animal and cellular models and proposed to be part of the pathological process. Moreover, dFil has been shown to accumulate in insoluble aggregates in *Drosophila* muscles when its autophagic protein turnover was impaired (Brooks et al., 2020). Consequently, we also studied the distribution of dFil in both control and KI mutants. In control flies, dFil was found as expected at the Z-disc, but also in aggregate-like dense bodies interspersed between actin filaments (Fig. 3D). We quantified the number and size of these puncta but failed to observe any differences between controls and dFil mutants (Fig. 3D; Fig. S6). Thus, the three variants do not affect neither cardiac function nor sarcomeric organisation and do not seem to lead to filamin aggregation in flies.

### Deletion of the dFil C-term region containing the last four Ig domains do not affect cardiac function or sarcomeric integrity

**Fig. 4.**
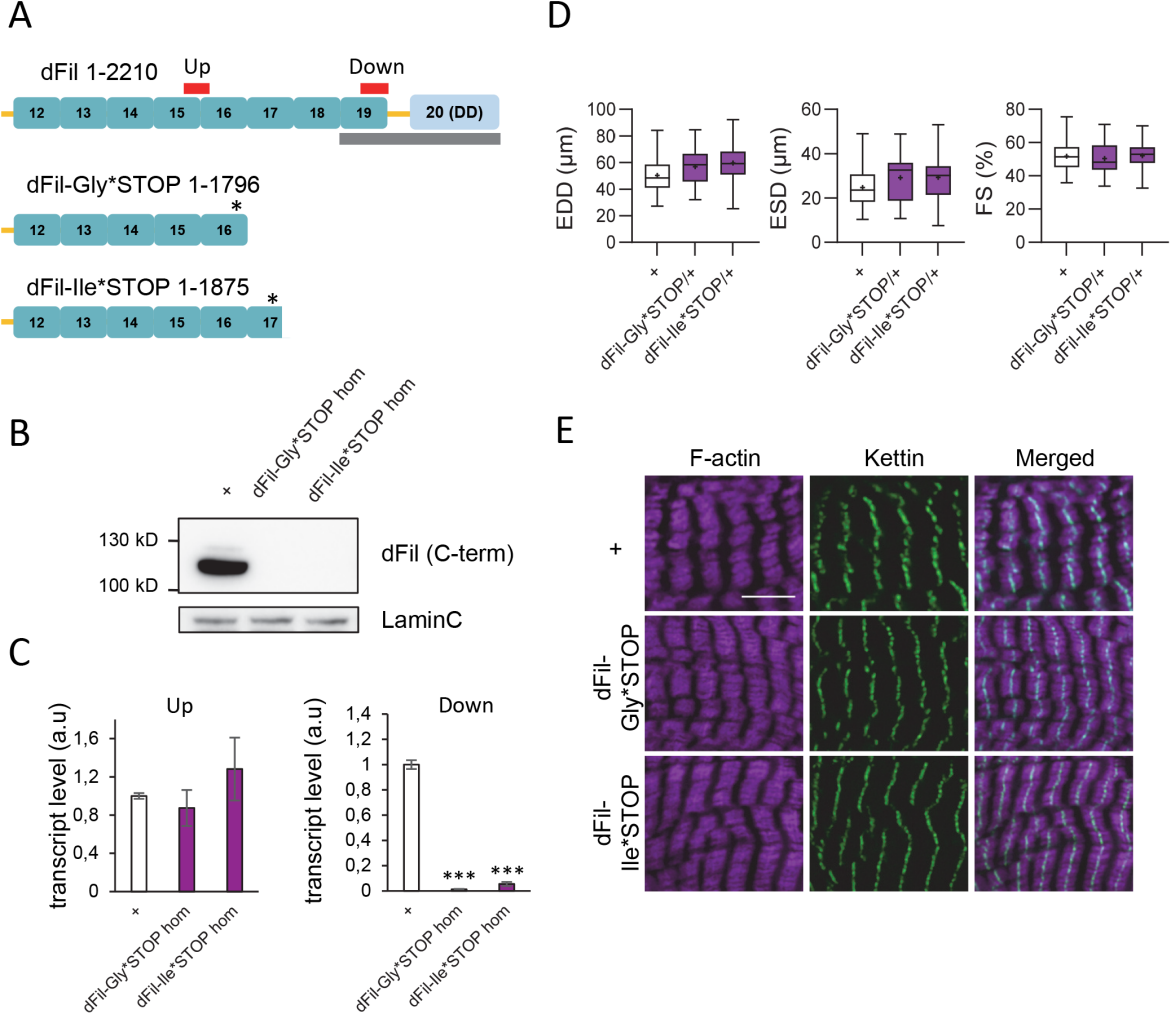
**Deletion of dFil C-term region do not affect cardiac function or sarcomeric organisation.** (A) Schematic diagram of dFil, dFil-Gly*STOP and dFil-Ile*STOP truncated proteins (isoforms Cher-PA). Immunoglobulin-like domains 12-20 are indicated. ABD: actin binding domain; DD: dimerization domain. The grey rectangle indicates the region recognized by anti-dFil antibody and the red rectangles the positions of amplicons used for transcript quantification. (B) The level of expression of dFil in 10-day-old control (*w^1118^*) flies compared to dFil-Gly*STOP and dFil-Ile* homozygous flies was analysed by western blot (WB), with antibody directed against LaminC as a loading control. (C) Quantification of *cheerio* transcripts. qRT-PCR were performed on whole RNA extracts of 10-day-old male flies with primers located upstream (Up) or downstream (Down) the position of the PiggyBac transposon. Error bars are s.e.m.; significant differences are indicated: *** *P*<0.001. (D) End-diastolic diameters (EDD, µm); end-systolic diameters (ESD, µm) and fractional shortening (FS, %) of 21-day-old *Hand-GS>UAS-mitoGFP controls* (+, *n*=69), *Hand-GS>UAS-mitoGFP*, dFil-Gly*STOP/+ (*n*=17) and Hand-GS>UAS-mitoGFP, dFil-Ile*/+ (*n*=19) male flies. No significant differences between genotypes were detected. (E) Hearts of 10-day-old adult control (*w^1118^*) and homozygous dFil-Gly*STOP and dFil-Ile*STOP male flies were dissected and double-labelled with phalloidin to stain F-actin and with an anti-Kettin antibody. Scale bar: 5 µm.

By the course of the generation of the dFil KI lines, intermediate lines were generated in which a PiggyBac transposon bearing a DsRed marker was introduced at close distance from the mutated codon. This led to the formation of two truncated proteins deleted from amino-acids 1797 for dFil-Gly*STOP and from amino-acid 1876 for dFil-Ile*STOP and dFil-Val*STOP lines, the indicated positions being given for dFil isoform A (Fig. 4A). Since the KI variants themselves were shown above to be non-pathogenic in flies, we considered that these truncated proteins were relevant to evaluate the impact of these C-term deletions on Filamin function. We checked by western blotting analysis the absence of detected protein using the anti-dFil antibody that recognises the C-term part of the protein. On extracts made from whole bodies of male flies, we were only able to detect the shorter 90 kD isoform which was not detectable in dFil-Ile*STOP and dFil-Val*STOP flies (Fig. 4B). A longer isoform of 240 kD is expressed mainly in ovaries (Sokol and Cooley, 1999). We performed western blots on ovary extracts and observed that this isoform, detectable in controls, was also not detectable in the dFil-Ile*STOP and dFil-Val*STOP ovaries, as expected (Fig. S7A). Then, we quantified the transcript levels by qRT-PCR using primers located upstream or downstream the positions of the transposon insertion (Fig. 4C). With the downstream primers, the levels of *cheerio* transcripts were barely detectable, as expected due to the presence of the SV40 polyA sequence located downstream the DsRed marker in the PiggyBac transposon. The upstream primers revealed similar levels of *cheerio* transcripts in dFil-Ile*STOP and dFil-Gly*STOP homozygous adult flies compared to controls, supporting the fact that the truncated proteins are expressed at a normal level. We first evaluated effects of the truncations on developmental viability. dFil-Gly*STOP homozygous flies are partly viable with a developmental lethality mainly occurring before the pupal stage. dFil-Ile*STOP flies are mainly viable, without prepupal lethality and 79% of pupae reaching the adult stage (Table S1). Median lifespan of males of both lines is only slightly lower than that of *w^1118^* control males (Fig. S7B). The longevity phenotype is more pronounced in females, mainly due to decreased survival in the first 3 weeks of adult life (Fig. S7C). In addition, these females are sterile (Table S1). Indeed, female sterility was the initial phenotype observed in the *cher^1^* mutant and has been related to the role of dFil in the assembly of ovarian ring canals, which most likely explain the sterility observed here (Robinson et al., 1997; Sokol and Cooley, 1999). Decreased survival of dFil-Ile*STOP mating females was much more pronounced compared to virgin females, suggesting that this phenotype is directly linked to ovarian dysfunction (Fig. S7D). To our knowledge, the molecular nature of the *cher^1^* mutant is not known, but it has been shown that the long 240 kD isoform was absent in these flies, whereas the short 90 kD isoform remained expressed (Sokol and Cooley, 1999). Combined with our results, this suggests that the presence of the full 240 kD isoform, containing both the N-term actin-binding domain and the C-term dimerization domain is required for ovarian function in female flies.

We then analysed cardiac functional parameters of *Hand-GS>UAS-mitoGFP* flies carrying dFil-Gly*STOP and dFil-Ile*STOP at the heterozygous state. EDD, ESD and FS were not significantly different from controls (Fig. 4D). In addition, sarcomeric organisation in cardiomyocytes of dFil-Gly*STOP and dFil-Ile*STOP homozygous flies failed to detect any structural defects (Fig. 4E). Thus, the C-term part of the protein including Ig17 to Ig20 is not required for cardiac function and sarcomeric maintenance while it is required for female fecundity.

## DISCUSSION

Considering the prevalence of FLNC variants in patients with HCM and DCM cardiomyopathies, it is of major interest to develop tools, including animal models, to assess their pathogenicity and to better understand the underlying pathophysiological mechanisms. Haploinsufficiency is a proposed mechanism for FLNC-related DCM, making it relevant to study the cardiac impact of FLNC depletion. Indeed, a cardiac-specific KO mouse has been recently generated, with complete loss of the protein leading to embryonic lethality, highlighting the importance of FLNC in cardiomyocyte development (Zhou et al., 2020). By using an inducible system to knockout FLNC specifically in young adult mice, progressive cardiac dilatation and extensive fibrosis were also observed. In Zebrafish, injection of embryos with a morpholino targeting *flncb*, one of the two *FLNC* orthologues, also induced cardiac dilatation (Begay et al., 2016). Our approach is complementary to these models and provides an opportunity to study the impact of developmental versus adult-specific filamin depletion on heart function, the RU486-inducible system also allowing the control of dFil level. Notably, adult-specific depletion led to cardiac dilatation and impaired systolic function, further confirming the role of dFil/FLNC in the maintenance of cardiac function at the adult stage. We have also characterised the sarcomeric apparatus and observed a strong disorganisation of the actin network associated with a widening of the kettin band, a phenotype that has also been observed in *Drosophila* indirect flight muscles (González-Morales et al., 2017). When dFil depletion was induced only during adulthood, the widening of the kettin band was progressive, showing that dFil is required in the heart for the maintenance of sarcomere integrity and Z-disk stability at the adult stage.

Surprisingly, deletions of the C-term domain of dFil, containing up to the last four Ig-like domains 17-20, did not affect sarcomeric organisation of cardiomyocytes at the homozygous state, or cardiac function at the heterozygous state. dFil Ig-like domain 20 is well conserved, presenting 52% of identity with the corresponding hFLNC Ig-like domain 24. This domain is involved in the protein dimerization, which is thought to be crucial for FLNC actin-crosslinking function. Here, we show that this domain is required for female fecundity but dispensable for viability and cardiac function. Interestingly, this has also been recently suggested in mammals. The p.W2711X mutation removes the last 16 amino-acids of mouse FLNC, impeding the formation of dimers through Ig-like domain 24. Mouse homozygous for this mutation are viable, with a relatively mild myopathy, meaning that dimerization through this domain is not strictly required for primary myogenesis (Schuld et al., 2020). Moreover, human FLNC presenting the same 16 amino-acid C-term deletion, expressed in Zebrafish depleted for endogenous FLNC, is able to localise correctly at the Z-disk and to rescue fiber disintegration (Ruparelia et al., 2016). Altogether, these results suggest either that dimerization is not strictly required for dFil/FLNC function, or more likely the existence of a more amino-terminal alternative dimerization domain. Indeed, Ig-like domains 18-21 of hFLNC, with a strict requirement of Ig20, were shown to dimerize by yeast two-hybrid experiments and cross-linking assays (Schuld et al., 2020). Thus, it is possible that the corresponding Ig-like domains of dFil (Ig14 to Ig17) have similar properties, explaining the viability of flies expressing the truncated dFil-Gly*STOP and dFil-Ile*STOP proteins. This part of our study points the interest of using *Drosophila* to identify *in vivo* essential versus dispensable Ig-like domains of dFil/FLNC. This could be assessed in the future by generating flies with CRISPR/Cas9-induced internal deletions, providing useful information for potential long-term therapeutic applications based on exon-skipping strategies, in particular for patients carrying truncating mutations.

Missense variants have been associated to the development of HCM in several patient cohorts (Eden and Frey, 2021; Verdonschot et al., 2020). However, there is only limited data concerning the impact of these variants on cardiac function. In this study, we aimed to use *Drosophila* as an *in vivo* animal model to assess the pathogenicity of selected variants, located in the Rod2 domain. Indeed, HCM-associated missense variants are clustered in this domain, in particular in Ig20-22. The Rod2 domain contains binding sites for numerous partners (Mao and Nakamura, 2020), suggesting that perturbations of interactions with some of them might be involved in the pathogenicity of these variants. However, we failed here to reveal a cardiac functional phenotype either in heterozygous of homozygous KI lines. Several HCM-associated missense variants have been shown to promote FLNC aggregation in a cellular model (Valdés-Mas et al., 2014), one of which, A2430V located in Ig22, having been recently shown to promote aggregates formation in myocardial tissue of a patient (Schanzer et al., 2021). Moreover, the Ig15-18 of dFil (corresponding to hFLNC Ig19-22) has recently been involved in an interaction with the kinase NUAK which was required together with Starvin/BAG3 for dFil autophagic degradation (Brooks et al., 2020). This prompted us to study the distribution of dFil in our KI flies. We observed formation of aggregate-like bodies in cardiomyocytes, but their number and size were not significantly different between KI flies and controls, showing that the variants do not increase the propensity of dFil to aggregate. Altogether, these results argue against the pathogenicity of these variants. However, negative results in this context are subject to various interpretations, and we cannot exclude the lack of effects being due to unconserved physio-pathological mechanisms for these specific variants or unconserved proteins interacting with FLNC in the Rod2 domain. Although it did not allow here to confirm pathogenicity, we believe this *Drosophila* KI approach, which is relatively fast and low-cost compared to mammalian models, to be of major interest, providing the opportunity to study *in vivo* the effects of variants expressed at endogenous levels. To our knowledge, this study constitutes the first gene editing strategy in *Drosophila* to evaluate the cardiac pathogenicity of human variants and paves the way for future cardiac functional screening of human variants in flies.

## MATERIALS AND METHODS

### *Drosophila* stocks and culture methods

UAS-mitoGFP (*w[1118]; Pw[+mC]=UAS-mitoGFP.AP2/CyO*), UAS-Cher-RNAi1 (*y[1]v[1]; P{TRiP.JF02077}attP2*), UAS-Cont-RNAi1 (*y[1]v[1];P{y[+t7.7]v[+t1.8]=TRiP.JF03047}attP2*) and MHC:GFP (*y[1]w;PBac{HpaI-GFP.A}MhcYD0783*) lines were obtained from the Bloomington Stock Center. *w[1118],* UAS-Cher-RNAi2 (*P{KK107518}VIE-260B*, v107451) and UAS-Cont-RNAi2 (*P{GD10097}*, v21206) were obtained from the Vienna *Drosophila* Resource Center. The HandGS GeneSwitch line was described in (Monnier et al., 2012). The fly food used for all experiments contained 82.5 mg ml^−1^ yeast, 34 mg ml^−1^ corn meal, 50 mg ml^−1^ sucrose, 11.5 mg ml^−1^ agar, and 27.8 μl ml^−1^ methyl 4-hydroxybenzoate (stock solution 200 g L^−1^ l in ethanol). When required, RU486 (Betapharma) was incorporated in the fly food from a 20 mg ml^−1^ stock solution in ethanol. Adult flies were collected within 24 h of pupal eclosion under brief CO2 anaesthesia, housed in groups of 20–30, under a 12 h:12 h light:dark cycle and transferred every 2 to 3 days onto fresh food medium prior to cardiac imaging or dissections for immunostaining experiments.

### Generation of dFil KI and dFil-STOP lines

CRISPR mediated mutagenesis was performed by WellGenetics using modified methods of Kondo and Ueda (Kondo and Ueda, 2013). In brief, gRNA sequences [GTCCTACAAGGTCACCGAAC(CGG) for dFil-Ile* and dFil-Val* and TATTCCGAGATGCACATACC(CGG) for dFil-Gly*] were cloned into U6 promoter plasmids. Cassettes I1849T, V1865L or G1789S PBacDsRed containing two PBac terminals and 3xP3 DsRed and two homology arms with the point mutations were cloned into pUC57 Kan as donor templates for repair. *cheerio* targeting gRNAs and hs Cas9 were supplied in DNA plasmids, together with donor plasmids for microinjection into embryos of control strain *w[1118]*. CRISPR generates a break in cheerio and is replaced by cassettes I1849T, V1865L or G1789S PBacDsRed, which introduce a STOP codon 8 amino acids after AA 1875 of dFil (coordinates of isoform Cher-PA, Flybase ID FBpp0088478) for the I1849T and V1865L cassettes and 7 amino acids after AA 1796 for the G1789S cassette. F1 flies carrying selection marker of 3xP3 DsRed were further validated by genomic PCR and sequencing and subsequently called dFil-Ile*STOP, dFil-Val*STOP and dFil-Gly*STOP. Next, the PBacDsRed was precisely excised using the piggyBac transposase driven by the alphaTub84B promoter provided by the w*; CyO, P{TubPBac T}2 / wgSp 1 line leaving only one silenced TTAA sequence embedded in the coding region of *cheerio* in dFil-Ile*, dFil-Val* and dFil-Gly* lines. The sequences of the three lines have been confirmed by Sanger sequencing after DNA genomic extraction on whole flies using standard procedures. PCR (Tm 60°C, 34 cycles on BioRad Thermocycler, Promega amplification reagents) have been performed using specific primers (forward: CGCGATGCTGTTCCAATCAC and reverse: TTCCGTTCTCGCGTGGATAG). After PCR purification (Qiagen PCR purification kit), Sanger sequencing have been realised with the forward primer (Eurofins genomics).

### *In vivo* functional heart analysis

The functional heart analysis was performed as described in Monnier et al., 2012. Flies were anesthetised with FlyNAP (Carolina Biological Supply Company). The anterior parts of heart (abdominal segments A1/A2) were observed with a Zeiss SteREO Lumar.V12 Stereomicroscope, with a NeoLumar S 1.5× objective. Video movies were acquired with an Hamamastu Orca Flash 4.0 LT camera (50 frames per second, 501 frames per movie). Each video was analysed as described in (Monnier et al., 2012) to extract EDD, ESD and FS. FS was calculated as described in (Fink et al., 2009). Results are presenting as boxplots extending from the 25th to 75th percentiles, with the horizontal bar at the median,+for the mean and the whiskers showing the most extreme data points. Statistical significance was assessed by one-way Anova followed by *post-hoc* Tukey analysis for multiple comparisons.

### Immunostaining of adult *Drosophila* hearts and image analysis

Dissection and immunostaining were performed as described in (Monier et al., 2005). Six to ten hearts were dissected for each independent condition. The following primary antibodies were used at 1/500: rabbit anti-GFP (TP401, Torrey Pines), rabbit anti-dFil (directed against the 227 last dFil amino-acids, first kindly provided by Erika Geisbrecht and next by Boster Bio) and rat anti-kettin (Ab50585, Abcam). The secondary antibodies goat anti-rat (A-11006, Abcam) and goat anti-rabbit (A-32731, Abcam) conjugated with Alexa Fluor 488 dye were used at 1/500. F-actin was stained with Phalloidin–Atto 647N (Merck) at 1/500. Samples were mounted onto slides in ProlongGold ProLong™ Gold Antifade Mountant (ThermoFisher Scientific). Images were taken onto a Zeiss LSM980 spectral Airyscan 2 at ImagoSeine Imaging platform. Images were processed using Fiji. For quantification of the width of the kettin band, three different regions of the A1/A2 heart region were analysed for each heart. A line spanning 6 to 8 sarcomeres was drawn and the intensity of the kettin signal was extracted using the Plot Profile function in Fiji. The sarcomere length was defined as the distance between two successive peaks of signal intensity. Quantification of aggregates was performed using the Analyse particle function in Fiji, with a selection of particles with a surface greater or equal to 0.05 µm^2^. Statistical significance was assessed with non-parametric Mann–Witney–Wilcoxon tests.

### Western blot analysis

Whole flies (ten per sample) were frozen at −20°C and lysed at 4°C in 300 µl of Laemmli Buffer supplemented with 1% Complete protease inhibitor (Roche) and NuPAGE™ Sample Reducing Agent (Thermo Fisher Scientific). Sample input was normalised on fly's weight. Proteins were separated by SDS-PAGE on 4–12% polyacrylamide gradient gels (ThermoFisher Scientific) and then electrotransferred onto nitrocellulose membranes (Bio-Rad). Standard immunochemistry protocols were used with primary rabbit anti-dFil (1/5000, provided first by Erika Geisbrecht and next by Boster Bio) and mouse anti-LaminC used as a loading control (LC28.26, 1/10,000, DSHB) and rabbit and mouse HRP secondary antibodies (Jackson ImmunoResearch Laboratories, ref 111-035-144, and ref 111-035-003 respectively, 1/50,000). Immunoreactivity was imaged and quantified with ImmobilonTM Western Chemiluminescent HRP Substrate (Merck Millipore) on an Amersham Imager 600 apparatus.

### Quantification of transcripts by qRT-PCR

RNA extractions were performed as described in (Reinhardt et al., 2012) and treated with dsDNase (ThermoFisher Scientific) according to the manufacturer's instructions. cDNAs were synthetised from isolated total RNA samples using SusperScript™III Reverse Transcriptase (ThermoFisher Scientific). qPCRs were performed with the qPCR Mix (Promega) on a LightCycler480 (Roche). The ribosomal gene *rp49* was used as an internal reference for normalisation. The primers used for amplifications of *cheerio* transcripts were 5′- GGGCAAGCACATCAACA-3′ and 5′CATGGGTTTGACCCTCC-3′, for the upstream primers and 5′-CATCCGCTAACTTGTCG-3′ and 5′- GGAGGGCTACAAGGTCC −3′, for the downstream primers. *rp49* gene: 5′-CCGCTTCAAGGGACAGTATCT-3′ and 5′-CACGTTGTGCACCAGGAACTT-3′. Quantifications were made on three to four independent biological samples with four technical replicates for each biological sample. Statistical significance was assessed by unpaired *t*-test.

### Viability and fecundity measurements

Prepupal viability is given as the percentage of homozygous individuals reaching the pupal stage, estimated by counting the number of heterozygous [marked with the tubby (tb) dominant phenotypic marker] versus homozygous pupae in the progeny of dFil-Gly*STOP/TM6tb or dFil-Ile*STOP/TM6tb flies. Pupal viability was estimated by counting the number of homozygous pupae reaching the adult stage. Female fecundity is given as the percentage of females with viable progeny, estimated by crossing females individually with four wild-type males.

### Lifespan experiments

Male and virgin female flies were collected within 24 h of eclosion under brief CO_2_ anaesthesia, housed in groups of 30, and raised at 26°C under a 12 h:12 h light:dark cycle. They were transferred every 2 days on fresh food, and dead flies were counted. For lifespan experiments on mated females, females were allowed to mate with males for 4 days and then collected and housed in groups of 30.

### Statistical analysis

All statistical tests were assessed using Prism software (GraphPad, PrismV6.01).
